# Anal Canal Duplication Mimicking Recurrent Abscess: A Case Report and Review of the Literature

**DOI:** 10.3389/fsurg.2022.908390

**Published:** 2022-05-12

**Authors:** Dandan Li, Shuaibin Liu, Jiexiong Feng, Jixin Yang

**Affiliations:** ^1^ Department of Pediatric Surgery, Tongji Hospital, Tongji Medical College, Huazhong University of Science and Technology, Wuhan, China; ^2^ Hubei Clinical Center of Hirschsprung’s Disease and Allied Disorders, Wuhan, China

**Keywords:** Anal canal duplication, crissum abscess, total surgical excision, secondary anal orifice, duplication of the gastrointestinal tract

## Abstract

**Background:**

Anal canal duplication (ACD) is a very rare duplication of the gastrointestinal tract and is described as a secondary anal orifice along the posterior side of the normal anal canal. Early surgical removal is advisable, also in asymptomatic patients, because of the risk of inflammatory complications, such as recurrent crissum abscess, and malignant changes.

**Case presentation:**

A previously healthy 2-year-old boy was evaluated in the emergency department with fever. He complained of anal pain in the absence of incentive. Physical examination and ultrasound confirmed a diagnosis of perianal abscess. He was treated with incision and drainage of the abscess and intravenous antibiotics. Two months after his discharge from the hospital, he developed fever and had intervals discharge pus and pain in the same locations. Colorectal endoscopy revealed that there was no fistula opening at the rectal wall. Intraoperative fistulography showed a fistulous tract that was connected to a subcutaneous cavity. Excision of the fistulous tract and wide drainage of the deep postanal space were performed. The patient was referred to our hospital for further evaluation 6 months later. Physical examination showed a secondary anus that had not been noticed before. MRI showed an anal fistula between 1 and 3 o’clock, and preoperative fistulography revealed a 3-cm-long tubular structure without any connection with the rectum. The diagnosis of ACD was made by intraoperative examination with a metal catheter and the postoperative pathological analysis. The duplicated anal canal was resected completely via a perianal approach without any rectal injury. Histology showed a squamous epithelium in the distal end with some smooth-muscle fibers. After a follow-up of 8 months, the patient has been doing well.

**Conclusion:**

Recurrent crissum abscess should raise clinical attention to alimentary tract congenital malformations such as ACD. Prompt recognition of these unique presentations of ACD is needed, and complete excision through a perineal approach or posterior sagittal approach is recommended.

## Background

Anal canal duplication (ACD) is a very rare duplication of the gastrointestinal tract and is described as a secondary anal orifice along the posterior side of the normal anal canal. ACD is usually detected incidentally in the first few years of life and is predominantly found in girls. Most ACDs end blindly, without connecting with the rectum, as in our case. The histologic features of ACD are squamous epithelium at the distal wall, transitional epithelium at the cranial wall, and smooth-muscle cells in the canal wall (1, 2). This report describes the rare case of a 2-year-old boy with ACD mimicking recurrent perineal abscess. We review the characteristics of this type of disorder and discuss the importance of early diagnosis and surgical treatment.

## Case Presentation

### First Hospital Course

A 2-year-old boy, without medical history, first presented to the emergency department with fever, which lasted up to 3 days, and chief complaints of pain. He had never complained of anal pain. The patient was taking acetaminophen. On presentation to the emergency department, his body temperature was 39.7°C. Fortunately, he presented normoxic with blood pressure, respiratory rate, heart rate, and oxygen saturation. The results of laboratory tests included a white blood cell (WBC) count of 18 × 10^10^/L, with 74.6% neutrophils, and a CRP level of 117.9 mg/dl. On physical examination, erythematous swelling and fluctuation were seen around the perianal area at 2 o’clock, which was 3 cm from the anus. He experienced severe pain on palpation of his crissum. Ultrasound showed significant fluid collection in the subcutaneous tissue, measuring 5.4 × 4.0 cm in diameter. The patient was admitted for an abscess incision and drainage procedure and received empirical IV antibiotics. His fever resolved and the inflammatory markers showed downward trending, with WBC 6.1 × 10^10^/L and CRP 55.9 mg/l. The results of bacterial wound cultures (aerobic and anaerobic) and blood cultures were negative. After 7 days of IV antibiotics, he was discharged from the hospital with a 2-week course of oral antibiotic therapy.

### Second Hospital Course

The patient was admitted to Wuhan Children’s Hospital 2 months after his first hospitalization. His history was notable for recurrent infection and intervals discharge pus in the same locations for the past month. These lesions improved with oral antibiotics and drainage but recurred every 1 week, with drainage noted. His parents then brought him to Wuhan Children’s Hospital for additional evaluation and treatment. Examination revealed one erythematous, fluctuant nodule in the perianal region at 2:00. Colorectal endoscopy was performed, and a fistula opening was not found at the rectal wall. With the urethral catheter indwelling, the patient was placed in the prone position, and a sagittal incision was made. During the operation, a fistulous tract was noted to be connected to a subcutaneous cavity after injecting colored dyes of methylene blue, which is used to establish the direction and openings of a fistula under minimal pressure. Excision of the fistulous tract, with placement of a seton, as well as wide drainage of the deep postanal space, was performed. The histologic evaluation of the biopsy tissue revealed granulation tissue, acute and chronic inflammation with histiocytic reaction, superficial fibrinopurulent exudate, and focal collections of neutrophils. Tissue cultures were negative for pathogenic bacteria, fungi, and mycobacteria. Patient recovery was uneventful, and he was discharged on the 15th postoperative day.

### Third Hospital Course

**Figure 1:**
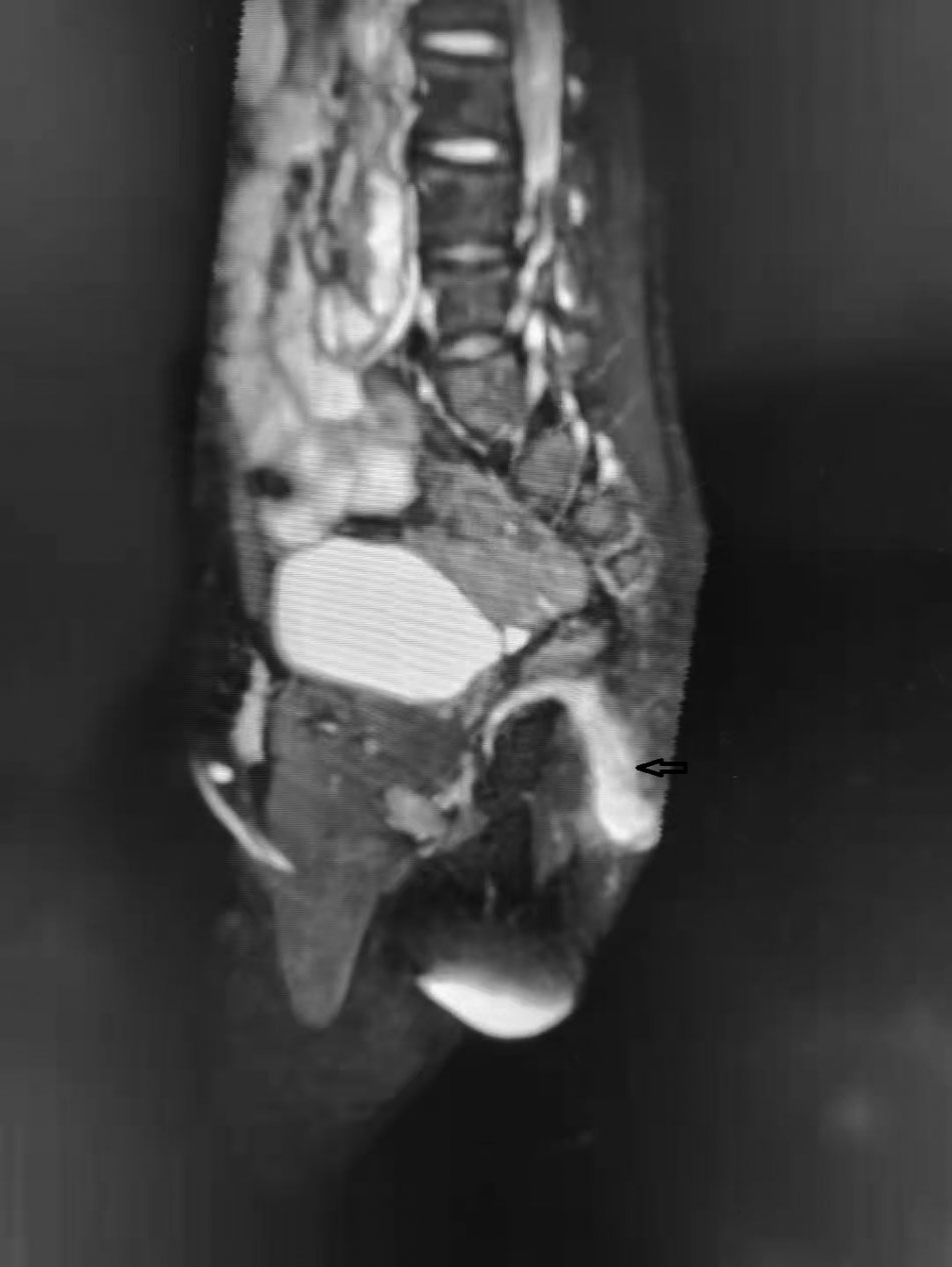
Sagittal T2-weighted perineum MRI shows a 25- mm anal fistula (arrow).

**Figure 2:**
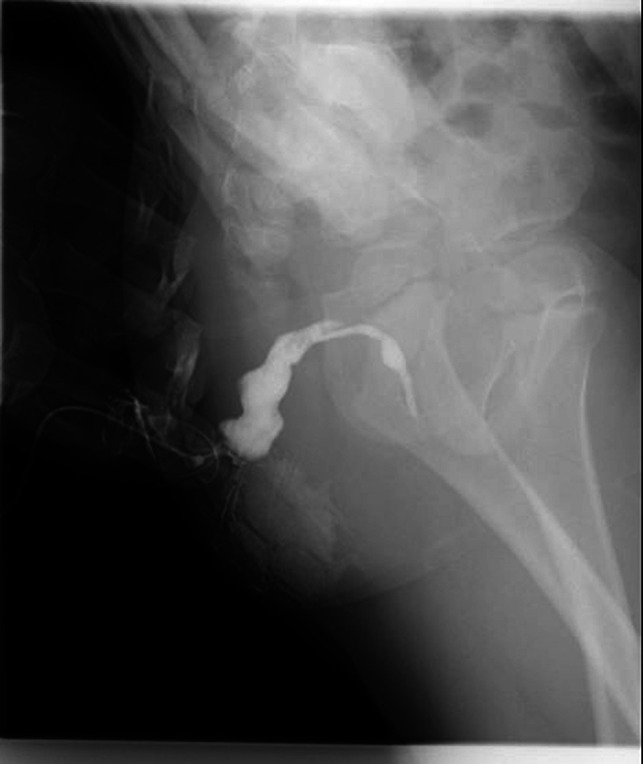
Fistulography reveals a 25-mm-long tubular structure of about 1–3 mm in width without any connection with the rectum.

The patient was referred to our hospital for the evaluation of recurrent fistula and pain at the same region 6 months after his last hospitalization. He had underwent two surgeries for perianal abscess previously at other medical facilities. Incisional drainage of the abscess resulted in a nonhealing perianal fistula. The fistula opening was situated about 2 cm at the anus at 2 o’clock. Intermittent purulent fluid was being discharged from the fistula. Digital rectal examination revealed no rectum fistula. Physical examination showed a small accessory opening, appearing as a secondary anus that had not been noticed before. Magnetic resonance imaging of the perineum showed an anal fistula between 1 and 3 o’clock in the lithotomy position (Figure 1). Meanwhile, there was a sinus tract that passed through the left gluteus maximus and communicated with the skin. Fistulography revealed a 25-mm-long tubular structure of about 1–3 mm in width without connection with the rectum (Figure 2). It was a wonder that the tubular structure had no communication with the second anal orifice. The clinical and radiologic features were consistent with secondary infection of an anal fistula.

**Figure 3:**
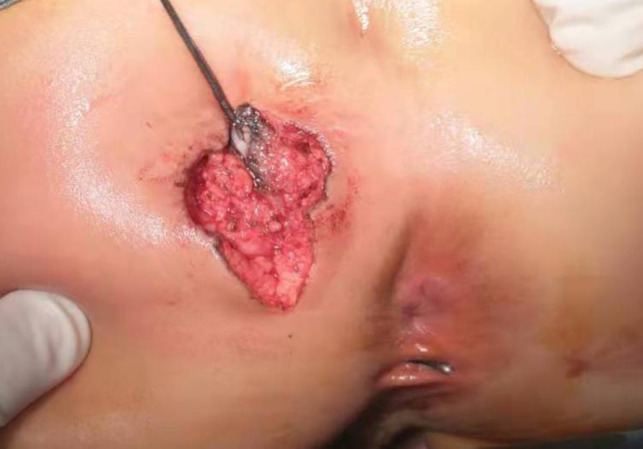
Perineal approach (prone position): the fistula had communication with the secondary anus, and this fact was checked with a metal catheter after intraoperative fistulography.

**Figure 4:**
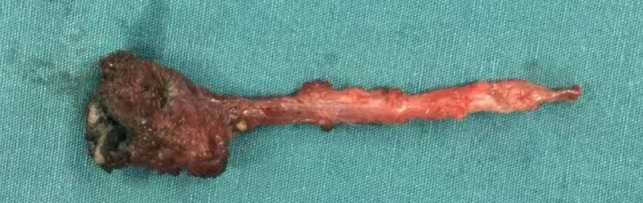
The resected specimen shows that the distal portion is a 40-mm-long duct.

**Figure 5:**
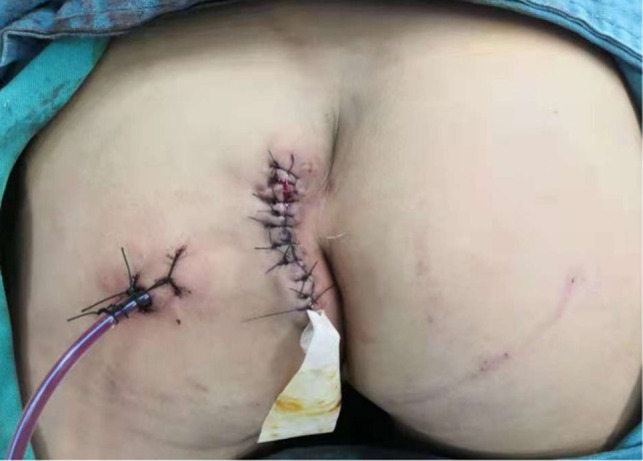
A hemovac drain is inserted, and the muscles are approximated with absorbable sutures.

**Figure 6:**
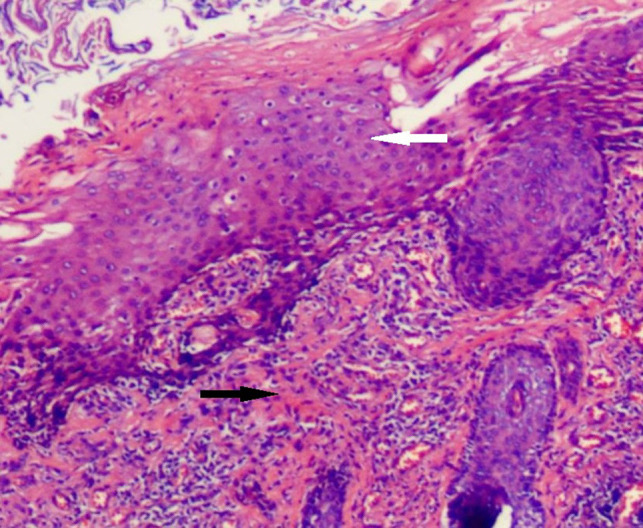
Histological findings (H&E). The duct is predominantly lined by squamous epithelium (white arrow) and smooth-muscle (black arrow).

**Figure 7:**
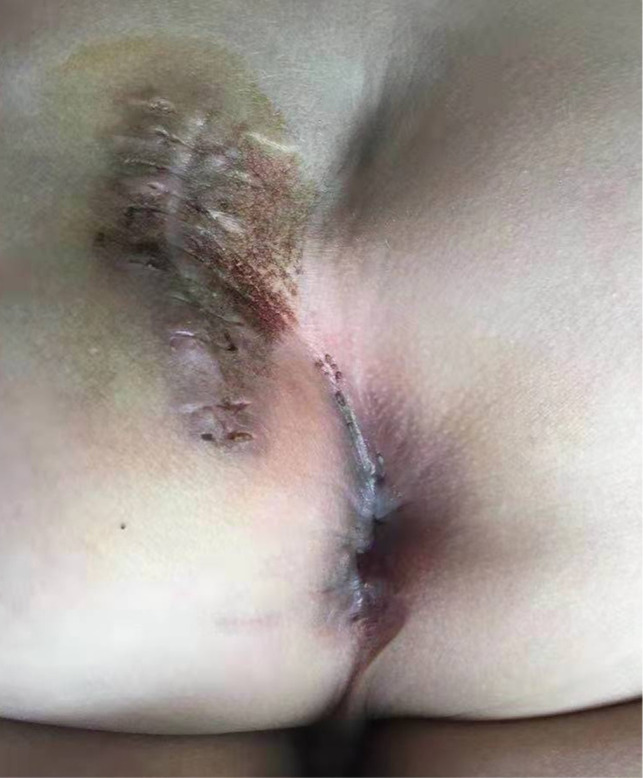
One month after surgery, the wound healed completely.

After the infection had been controlled, the fistula was excised by a perineal approach. Intraoperative fistulography under pressure showed that there was connection between the fistula and the secondary anus and was checked with a metal catheter (Figure 3). Fortunately, there was no connection between the fistula and the anal canal. The lesion circumscribing the distal tract through the left gluteus maximus was removed with incision from the perianal space, and the proximal tract, which extended from the accessory anal orifice to the left upper part of the perianal structures, was excised from the bottom up. Although the wall at the cranial end was partly shared with the rectum, it was resected completely without any rectal injury. The removed ACD had a narrow fistula, measuring approximately 2 mm in diameter and 40 mm in length (Figure 4). A hemovac drain was inserted, and the muscles were approximated with absorbable sutures (Figure 5). Squamous epithelium in the distal end and smooth-muscle cells lined the wall were demonstrated on pathology (Figure 6). One month after surgery, the wound healed completely (Figure 7). A follow-up of 8 months has shown no evidence of secretion passing from the opening of fistula, and the patient has been doing well.

## Discussion

ACD is the most less frequent gastrointestinal duplication. There are only 94 cases reported since its first description in 1956 by Dukes et al. (3). Abscess formation associated with ACD is a rare occurrence: only eight cases in children have been previously reported in the literature (4–8). Detailed information for all nine patients is given in Table 1.

**Table 1:** Summary of all reported anal canal duplication cases with abscess in the literature.

Cases	Year	Sex	Age (months)	Type	Communication with the anal canal	Associated anomalies	Length (mm)	Surgery	Complications
01	1997	F	27	Tubular	Yes	None	Unknown	Perineal approach	None reported
02	2003	F	41	Tubular	No	None	40	Posterior sagittal	None reported
03	2009	F	3	Cystic	No	Tethered cord Spina bifida occulta	50	Perineal approach	Anal injury repaired by simple closure and temporary bladder dysfunction and constipation
04	2010	F	1	Tubular	No	None	20	Posterior sagittal	None reported
05	2012	F	144	Cystic	Yes	None	40	Posterior sagittal	None reported
06	2016	F	168	Tubular	No	None	30	Perineal approach	None reported
07	2018	F	72	Cystic	No	None	17	Perineal approach and Posterior sagittal	Abscess formation
08	2019	F	156	Cystic	No	None	35	Posterior sagittal	None reported
09 (Present case)	2020	M	25	Tubular	No	None	40	Perineal approach	None reported

*M, male; F, female.*

ACD is characterized by an extra perineal orifice located right behind the normal anus at 6 o’clock, with a blind-ending tubular structure, not opening into the anorectum. According to the literature, more than half of the cases are reported to be asymptomatic (4, 9). Generally, the abnormality is discovered incidentally or during the careful perineum inspection at birth. ACD can occasionally become symptomatic after the age of 2 years. According to the documentary data, patients presenting with mild symptoms of diarrhea, constipation, anal pain, or mucous discharge accounted for 33%, and patients presenting with moderate symptoms such as recurrent fistulas, skin abscess, or sepsis accounted for 20%. In our case, a 2-year-old boy was diagnosed with ACD presented with a past history of recurrent episodes of abscess. Additionally, the severity of these symptoms actually appears to increase with aging at presentation. A total of 89.3% ACD are of tubular configuration, although they occasionally contain a cystic configuration (10.7%). Almost 30% cases had more than one additional anomaly. Additional anomalies include meningocele, presacral teratomas, or other presacral mass meningocele, congenital heart defects, cleft lip/palate, and genitourinary defects. There is a large female predominance. However, the reason for this predominance is unclear.

There are two hypotheses that have been proposed for the development of ACD. The embryologic basis for ACD remains unclear. Hamada et al. thought the pathogenesis of ACD might be explained by duplication of the dorsal cloaca in the early development stage (10). Another theory, proposed by Nievelstein et al., suggests that ACD can be defined as a late embryonic anomaly. In this theory, fetuses with a greater length of dorsal cloacal membrane than normal undergo a recanalization of the excess cloacal membrane to cause a duplicated anal canal (11).

The diagnosis can usually be clinically suspected by simple perineal inspection. In our case, a boy with recurrent crissum abscess was previously evaluated several times before ACD could be suspected and evidenced. Noninvasive methods such as fistulography, US, MRI, and pathologic findings are usually enough to diagnose ACD. Fistulography can reveal a tubular or cystic structure and its length and connection with the anal canal. Pelvic and abdominal ultrasound examinations are necessary to evaluate the presence of associated anomalies in neonates. MRI is routinely used in older children to detect associated anomalies such as sacrococcygeal teratoma and dermoid cyst. The diagnosis of our case was not easy, especially with recurrent episodes of perianal abscess and ignorance of the fact that he had two holes in the anus, which interfered with the doctor’s judgment. The diagnosis of ACD in our study was made by examination under general anesthesia with a metal catheter after suspicion by a simple perineal inspection. The exact diagnosis of ACD is based on histological examinations. Three characteristics are essential for histological diagnosis: (1) squamous epithelium in the caudal end, (2) transitional epithelium in the cranial end, and (3) smooth-muscle cells in the wall of ACD (11). Histological findings of our case were similar to those of the literature.

Total surgical excision is the recommended treatment for ACD, because it can cause infection risk due to the accessary gland structures and malignant changes (4, 7, 12). Early surgery may well prevent inflammatory complications such as abscess formation. In our case, the patient with ACD had complications with recurrent crissum abscesses and fistulas, necessitating more invasive treatments. The majority of patients received a complete removal via a perianal or posterior sagittal approach, which is both safe and effective. According to the literature, only one case of recurrence after the perineal approach has been described by Nakata et al. Care must be taken to avoid injury to the normal gut during complete ACD excision and the anal sphincter, which may potentially disrupt normal bowel function. Some authors suggest simple mucosal stripping of ACD, which is located very close to the anal canal. Surgical treatment usually results in good prognosis and minor surgical sequelae.

Information about patients of ACD with abscess is extremely limited in the reported literature. In all cases of ACD with abscess, except the present one, the other eight patients were female, and the average age of presentation was 76.5 months (range, 1–156 months). Five cases were tubular structures and four cases had a cystic form, which seemed inconsistent with the theory that most cases are tubular (13). Internal communication with the native anal canal was present in two patients. Although one patient had additional anomalies, common anomalies such as sacral tumors or anal stenosis were otherwise not found. The median length of the fistula was 34 mm (minimum, 17 mm; maximum, 50 mm). Surgical methods included a perineal approach for five cases and a posterior sagittal approach for five cases. Recurrence abscess formation after the perineal approach occurred only in case 7.

Survey such as that conducted by Mirzaei et al. has reported that three among four adult cases of ACD (age 20–50 years) presented with recurrent crissum abscess and fistula (8). The fact that none of them had communication with the anal canal indicated that the cause of abscess formation is not likely to be related with internal connection with the rectum. There are no rigid guidelines regarding the operative methods for ACD with abscess formation. In our experience, the patient underwent incision and drainage of the abscess, excision of the fistulous tract, and complete removal of the duplicated anal canal via the perianal approach. However, there is a limit to the depth that can be examined using the perineum approach. If removal is incomplete, then the recurrence of the abscess remains possible. Koga et al. and Jacquier et al. recommended the posterior sagittal approach for abscessed ACD surgery (5, 14).

## Conclusion

In the case of recurrent abscess formation and a posterior perineal orifice, ACD should be considered in the differential diagnosis. Surgical removal is recommended in early life, even in asymptomatic patients, because of the risk of recurrent abscess formation.
